# Infective endocarditis and relationship with diabetes mellitus - Patient characteristics, microbial etiology and mortality

**DOI:** 10.1007/s15010-025-02624-7

**Published:** 2025-09-10

**Authors:** Andreas Dalsgaard Jensen, Lauge Østergaard, Peter Laursen Graversen, Katra Hadji-Turdeghal, Jeppe K. Petersen, Peter Rossing, Christian Selmer, Jonas Agerlund Povlsen, Marianne Voldstedlund, Henning Bundgaard, Claus Moser, Emil Loldrup Fosbøl

**Affiliations:** 1 The Department of Cardiology, Copenhagen University Hospital, Rigshospitalet, Inge Lehmanns Vej 7, 16th floor, Copenhagen, 2100 Denmark; 2 The Department of Clinical Medicine, University of Copenhagen, Copenhagen, Denmark; 3 Steno Diabetes Center Copenhagen, Herlev, Denmark; 4 The Department of Endocrinology, Bispebjerg and Frederiksberg University Hospital, Copenhagen, Denmark; 5 The Department of Cardiology, Aarhus University Hospital, Aarhus, Denmark; 6 The Department of Clinical Microbiology, Copenhagen University Hospital Rigshospitalet, Copenhagen, Denmark; 7 Department of Clinical Microbiology, University of Copenhagen, Copenhagen, Denmark; 8 The Department of Immunology and Microbiology, University of Copenhagen, Copenhagen, Denmark

**Keywords:** Infective endocarditis, Diabetes mellitus, Epidemiology, Microbiology, Mortality

## Abstract

**Purpose:**

Infective endocarditis (IE) has been associated with severe outcomes when complicated by diabetes mellitus (DM). We aimed to report characteristics, microbial etiology, and mortality for patients with IE stratified by DM from a nationwide cohort.

**Methods:**

We used Danish registries, and patients with first-time IE (2010–2020) were stratified by DM. We computed inverse Kaplan-Meier estimates for one-year mortality from admission. We computed multivariable adjusted Cox regression for the adjusted one-year mortality from admission and discharge.

**Results:**

We identified 6,211 patients with first-time IE; 1,503 (24.2%) with DM (26.1% Type 1 DM, 68.1% male, median age 72.7 years); 4,708 (75.8%) did not have DM (67.0% male, median age 72.4 years). Patients with IE and DM had a higher proportion of chronic kidney disease (35.9% vs. 11.1%). The most predominant microorganism was *Staphylococcus aureus (S. aureus)* for patient with IE and DM (36.5%), and *Streptococcus* species (spp.) for those without DM (29.4%). Patients with IE and DM were associated with an increased one-year mortality from admission (41.1% [95% CI: 38.5%-43.6%] vs. 31.0% [95% CI: 29.6%-32.3%]). The adjusted mortality estimates were higher for patients with IE and DM compared to those without DM one year from admission (HR = 1.15 [95% CI: 1.04–1.27]), and one year from discharge (HR = 1.26 [95% CI: 1.09–1.46]).

**Conclusion:**

Patients with IE and DM were associated with a higher burden of kidney disease, *S. aureus* as the predominant microorganism, and increased one-year mortality both from admission and discharge. These findings call for improved management of IE in patients with DM.

**Supplementary Information:**

The online version contains supplementary material available at 10.1007/s15010-025-02624-7.

## Graphical Abstract

Created in BioRender. Jensen, A. (2025) https://BioRender.com/jok3rtl

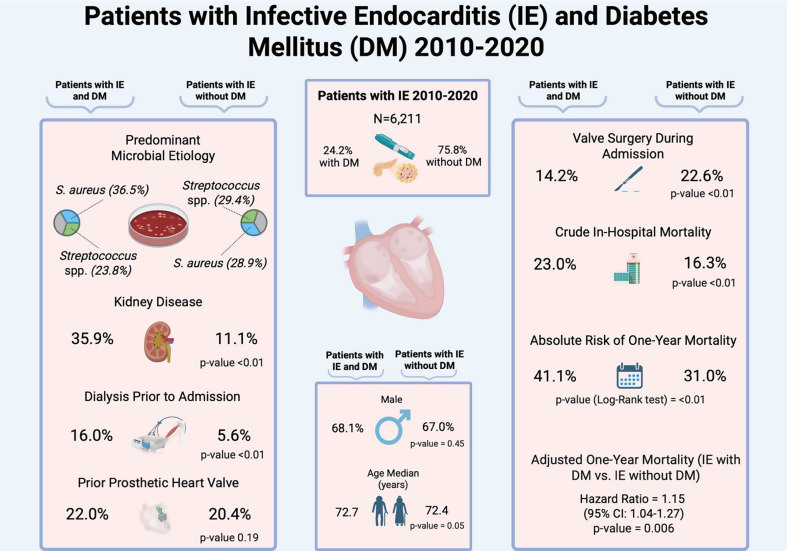

## Introduction

Infective endocarditis (IE) is a serious condition with high morbidity, high mortality, and a doubling of incidence rates in several western countries over the past decades [1–3]. The prevalence of diabetes mellitus (DM) in patients with IE has been reported between 12.0% and 34.4% and several studies have reported an increasing trend including in Denmark where the proportion rose from 11.1% in 1997 to 21.7% in 2017 [4–15]. Also, *Staphylococcus aureus* (*S. aureus*) is now the predominant microbial etiology for patients with IE [16–18]. Infections, including bloodstream infections, occur more often in patients with DM and they contribute to their excess mortality [19, 20]. The mechanisms are not fully understood, but patients with DM are associated with an altered efficiency of the innate immune system and a state of chronic inflammation [20]. Diabetic foot ulcers are a common complication in DM and they are associated with *S. aureus* infections [20, 21]. Also, patients with DM are associated with complex comorbidities such as kidney disease secondary to micro- and macrovascular changes [22]. The current guidelines on the management of IE have no specific recommendations for this large and clinically important group [1]. However, outcomes for patients with IE and DM have been reported as more severe compared to patients without DM, and longer duration of DM has been associated with an increased incidence of IE [11, 23]. We hypothesized that patients with DM may constitute an important and distinct group of patients with IE. The aim of this cohort study was to report characteristics, microbial etiology, and mortality for patients with IE stratified by DM in a nationwide setting, as this knowledge can help understand the challenges that remain in their treatment.

## Methods

### Data sources

We used Danish nationwide registries for this cohort study conducted for the period January 1st, 2010, until December 31st, 2020.

Every person born or residing in Denmark is assigned a unique identifier for personal identification (CPR-number) which is linkable across registries [24]. We used the following registries: (1) The Danish Civil Registration database, (2) The Danish National Patient Registry, (3) The Danish National Prescription Registry, (4) The Danish Microbiology Database, (5) The Danish Register of Causes of Death. The Danish Civil Registration database holds information on vital status, emigration, immigration, date of birth, sex, and several other variables [24]. The Danish National Patient Registry holds information regarding every hospital admission in Denmark since 1977 [25]. It is mandatory for the discharging physician to report information regarding the date of admission, the day of discharge, the primary diagnosis, and it is possible to add any secondary diagnoses at the time of discharge [25]. The Danish National Patient Registry was organized by the International Classification of Diseases 8th revision (ICD-8) from 1974 to 1993 and has been organized by ICD-10 since 1994. Surgeries are also reported to The Danish National Patient Registry and have been organized in accordance to the Nordic Medico-Statistical Committee Classification of Surgical Procedures (NOMESCO) since 1996 [25]. The Danish National Prescription Registry holds detailed information on all redeemed prescription medications dispensed at any pharmacy in Denmark since 1995 (including medication dispensed to persons living at nursing homes) [26]. The registry is organized by the Anatomical Therapeutic Chemical classification (ATC-codes). The Danish Microbiology Database (MiBa) contains information from every blood culture from every patient in Denmark since 2010 [27]. The Danish Register of Causes of Death holds information from the mandatory death certificates [28]. The Danish Register of Causes of Death holds information on date of death, manner of death, and cause of death as well as several other variables.

### Study population

**Fig. 1:**
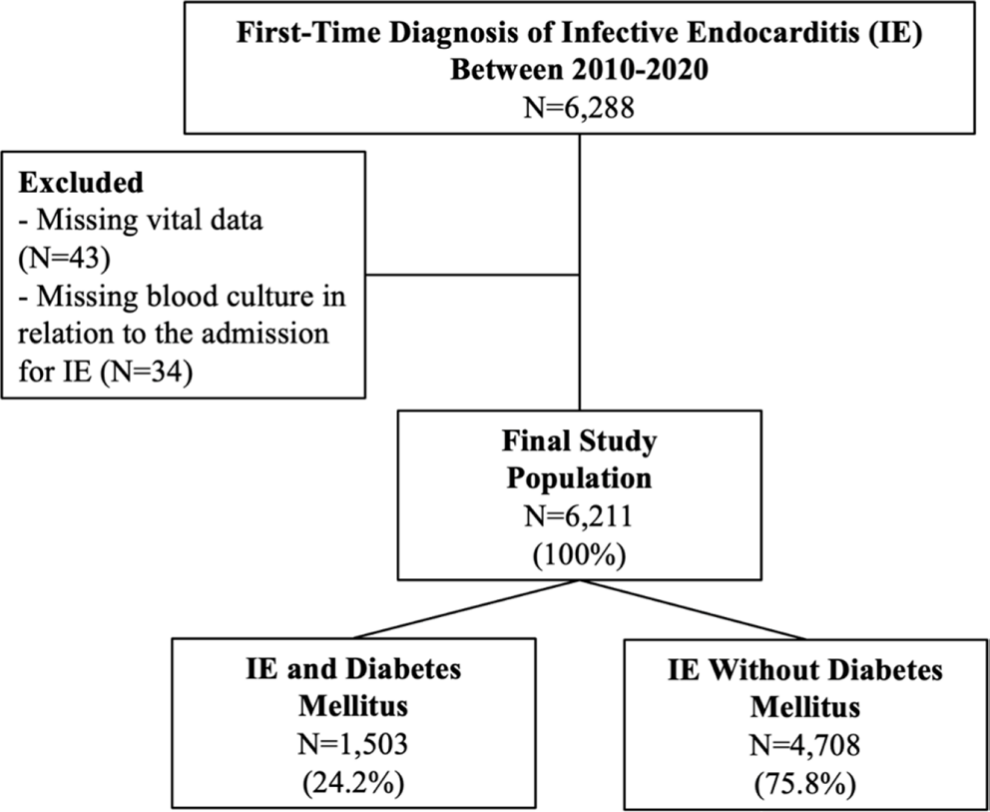
The figure shows the flowchart of patient selection with reason for exclusions listed. IE = infective endocarditis

Patients with IE were identified by ICD-8/10 codes, see Supplementary Table 1. We only included first-time events and if their first-time event was between 2010 and 2020. We used the following criteria: (1) Admission for 14 days or death within the first 14 days, (2) in-hospital diagnoses codes of either primary or secondary type. This approach has been validated by Ostergaard et al. who found a positive predictive value (PPV) of 90% for the diagnosis of IE when using the Danish registries [29]. Patients with missing vital data were excluded (*N* = 43), see Fig. 1.

We then stratified patients with IE by DM. We defined DM by one or both of the following: (1) a relevant ICD-8/10 code prior to IE admission, see Supplementary Table 1, (2) use of antidiabetic medication within 6-months prior to the IE admission, see Supplementary Table 1. Classifying patients as having DM by combining multiple registries is comparable to previous Danish studies [30–32]. We reported the proportion of Type 1 DM, see Supplementary Table 1. We did not consider patients who solely received Glucagon-Like Peptide-1 agonists (GLP-1) or Sodium-Glucose Co-Transport 2 Inhibitors (SGLT2i) as having DM (*N* < 4), as these have other indications too. Patients with an ICD-8 code of DM without a later ICD-10 code or use of antidiabetic medication within 6-months of IE were classified as patients without DM (*N* = 6). We performed supplementary analyses where patients with any type of DM were subdivided by use of insulins and where patients were subdivided by type of DM. Please see definitions of Type 1 DM, Type 2 DM, and use of insulins in Supplementary Table 1.

### Microbiology

We assessed blood cultures drawn within 30 days of the admission for IE and until discharge. We excluded those without blood cultures in this interval, as they were not considered to have IE (*N* = 34). The microbial etiology was grouped into the following: (1) *S. aureus*, (2) *Streptococcus* spp., (3) coagulase-negative Staphylococci (CoNS), (4) *Enterococcus* spp., (5) other (including HACEK-organisms [*Haemophilus*,* Aggregatibacter*,* Cardiobacterium*,* Eikenella*, and *Kingella*]), (6) blood culture negatives. We considered the type of microorganism in subsequent blood-cultures as the microbial etiology if the first blood culture was negative. We defined patients as blood culture negative if they had IE and blood cultures drawn in relation to their admission but none of them were positive.

### Baseline characteristics, follow-up, outcomes, and statistics

Baseline characteristics were presented in Table 1. Please see Supplementary Table 2 for the baseline characteristics when subdividing patients by use of insulins and Supplementary Table 3 for the baseline characteristics when subdividing patients by type of DM.

We assessed comorbidities any time prior to admission for IE, but the lookback for pharmacotherapy was 6 months. We assessed in-hospital and outpatient visits, and diagnoses of either primary or secondary type, see Supplementary Table 1.

Hypertension was defined by use of two or more anti-hypertensive medications, as done previously [33] or a by relevant diagnosis code, see Supplementary Table 1. Categorical variables were presented as counts and percentages, and continuous variables were presented as medians with 25th -75th percentiles. Differences between groups were assessed by chi-square test for categorical variables, and Kruskal-Wallis test for continuous variables. The outcome was all-cause mortality, and we reported both unadjusted and adjusted estimates of the following: (1) In-hospital mortality, (2) one-year mortality from admission, (3) one year mortality from discharge. We censured patients at the day of emigration if that occurred prior to the end of follow-up.

The following analyses were performed for in-hospital mortality: (1) Crude proportion of in-hospital mortality and chi-square method to test for statistically significant differences between groups, (2) unadjusted Cox regression modelling, (3) Cox regression adjusted for the following variables: Age, microbial etiology, sex, myocardial infarction, congestive heart failure, chronic obstructive lung disease, chronic kidney disease (those not dependent on dialysis), dependency of dialysis prior to admission, prior malignancy, prosthetic heart valve, and cardiac implantable electronic device (CIED).

The following analyses were performed for one-year mortality from admission and one-year from discharge: (1) Inverse Kaplan-Meier (1-KM) as estimates of absolute mortality risk and log-rank method to test for statistically significant differences between groups, (2) unadjusted Cox regression, (3) adjusted Cox regression with the same covariates as for in-hospital mortality. Valve surgery was added in an additional analysis of one-year mortality from discharge. We tested DM for interactions with sex, microbial etiology or age groups in the adjusted models, and found a statistically significant interaction for age groups. We performed further analyses to account for this, so that patients were stratified by their corresponding age median. We also performed the following supplementary analyses: (1) Patients with IE and DM were subdivided by use of insulins and we reported one-year mortality from admission, (2) patients with IE were subdivided by type of DM and we reported the one-year mortality from admission, (3) we subdivided patients by valve surgery during admission, and followed from either the day of surgery or the day of admission. Statistical analyses were conducted using SAS version 9.4, and R version 4.4.1 [34]. A p-value < 0.05 was considered statistically significant. Confidence intervals of 95% (95% CI) were reported when applicable.

## Results

In total, 6,211 patients with IE were included between 2010 and 2020, see Fig. 1. Of them 1,503 (24.2%) had IE and DM, and 4,708 (75.8%) had IE without DM. We found a similar distribution of males (68.1% and 67.0% respectively) and similar age medians (72.7 years vs. 72.4 years), see Table 1.

**Table 1:** Baseline characteristics

	First-time IE 2010–2020 *N* = 6,211 (100%)		
	**IE and DM** *N* = 1,503 (24.2%)	**IE without DM** *N* = 4,708 (75.8%)	p-value
**Demographics at Baseline**,** N (%)**			
Male	1,023 (68.1%)	3,155 (67.0%)	0.45
Age	72.7 (65.4, 79.3)	72.4 (61.5, 80.5)	0.05
Age Group			< 0.01
< 70 Years	562 (37.4%)	2,056 (43.7%)	
≥ 70 Years	941 (62.6%)	2,652 (56.3%)	
**Hospitalization and Procedures**,** N (%)**			
Days Admitted	38.0 (25.0, 49.0)	38.0 (28.0, 49.0)	0.34
Valve Surgery During Admission	213 (14.2%)	1,062 (22.6%)	< 0.01
**Comorbidities at Baseline**,** N (%)**			
Type 1 DM	393 (26.1%)	0 (0.0%)	< 0.01
Acute Myocardial Infarction	302 (20.1%)	473 (10.0%)	< 0.01
Congestive Heart Failure	518 (34.5%)	863 (18.3%)	< 0.01
Atrial Fibrillation/flutter	601 (40.0%)	1,219 (25.9%)	< 0.01
Disease of The Aortic Valve	481 (32.0%)	1,440 (30.6%)	0.30
Prosthetic Heart Valve	331 (22.0%)	962 (20.4%)	0.19
CIED	366 (24.4%)	733 (15.6%)	< 0.01
Ischemic/hemorrhagic Stroke	275 (18.3%)	587 (12.5%)	< 0.01
Chronic Obstructive Lung Disease	284 (18.9%)	521 (11.1%)	< 0.01
Liver Disease	153 (10.2%)	320 (6.8%)	< 0.01
Malignancy	286 (19.0%)	903 (19.2%)	0.90
Chronic Kidney Disease	539 (35.9%)	521 (11.1%)	< 0.01
Dependent on Dialysis	241 (16.0%)	262 (5.6%)	< 0.01
Hypertension	1,152 (76.6%)	2,198 (46.7%)	< 0.01
**Pharmacotherapy Within 6 Months Prior to Admission**,** N (%)**			
Statin	937 (62.3%)	1,530 (32.5%)	< 0.01
Aspirin	574 (38.2%)	1,114 (23.7%)	< 0.01
NSAID	227 (15.1%)	824 (17.5%)	0.03
Corticosteroids	203 (13.5%)	545 (11.6%)	0.05
Use of Insulins	576 (38.3%)	0 (0.0%)	< 0.01
Use of SGLT2-inhibitors or GLP-1 agonists	137 (9.1%)	< 4 (< 0.1%)	< 0.01
Use of other antidiabetic medication	810 (53.9%)	0 (0.0%)	< 0.01

IE = Infective Endocarditis, DM = Diabetes Mellitus, CIED = Cardiac Implantable Electronic Device, NSAID = Non-Steroidal Anti-Inflammatory Drug, SGLT2-inhibitors = Sodium-Glucose Co-Transporter 2, GLP-1 = Glucagon-Like Peptide 1 Agonists

Categorical variables presented as count and percent

Continuous variables presented as the median with corresponding 25th to P75th percentile

The proportion of patients who underwent valve surgery during admission was lower for patient with IE and DM (14.2%) compared to those without DM (22.6%). Patients with IE and DM had a significantly higher burden of comorbidities compared to patients without DM including chronic kidney disease (35.9% vs. 11.1%), chronic dialysis (16.0% vs. 5.6%), and hypertension (76.6% vs. 46.7%). There were 576 (38.3%) patients with IE and DM using insulins and 393 (26.1%) patients with Type 1 DM. These patients presented with even higher burden of comorbidities. Chronic kidneys disease was present in 50.9% of patients using insulins and this was 56.7% in patients with Type 1 DM, see Supplementary Tables 2 and Supplementary Table 3. Also, dependency of dialysis was more pronounced in both patients using insulins (22.2%) and patients with Type 1 DM (28.0%).

### Microbial etiology

**Fig. 2:**
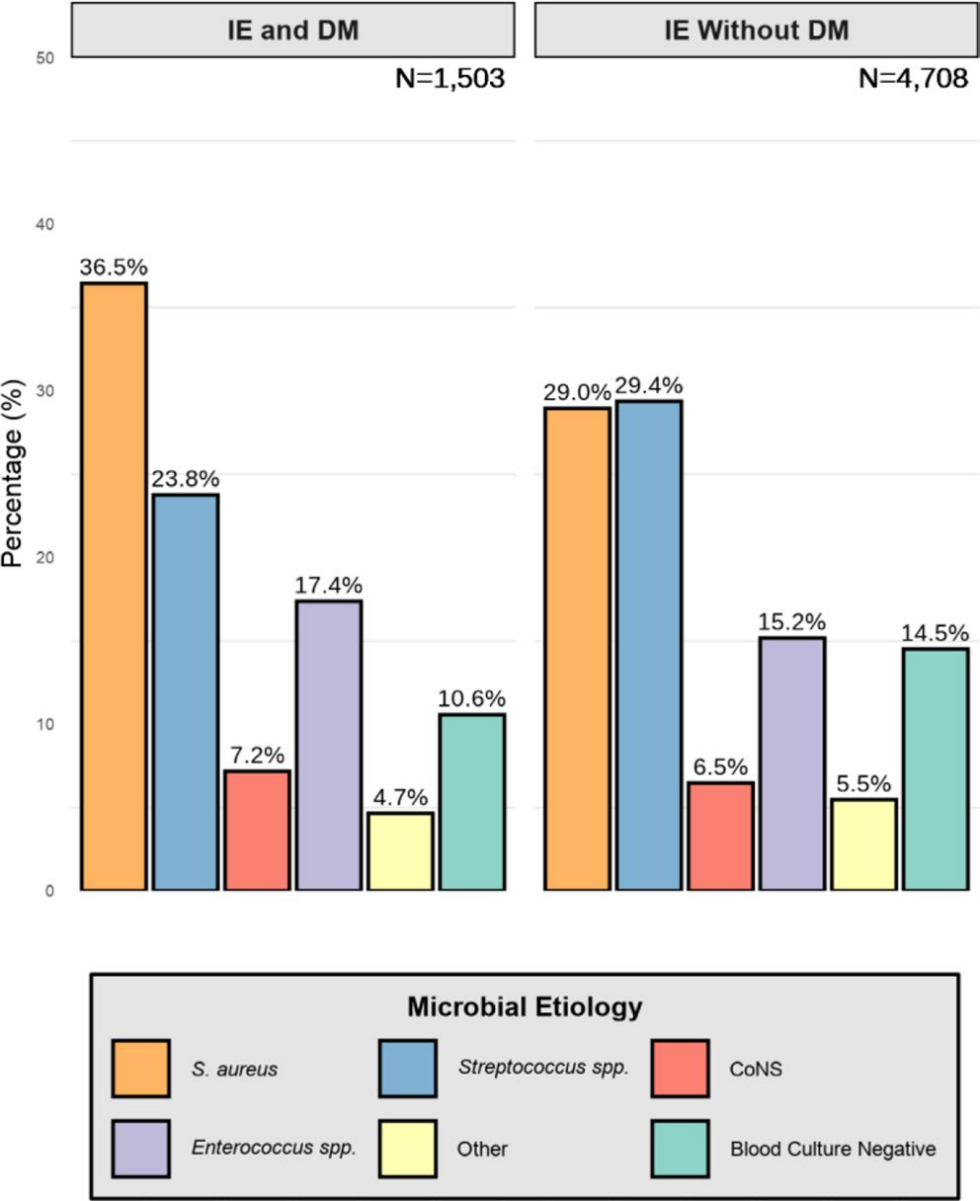
Microbial etiology in patients with infective endocarditis grouped by diabetes mellitus. The figure shows the microbial etiology in patients with infective endocarditis (IE) and diabetes mellitus (DM) compared to those without DM. IE = Infective Endocarditis, DM = Diabetes Mellitus, CoNS = Coagulase-Negative Staphylococci.

In the entire cohort of patients with IE the predominant microbial etiology was *S. aureus* (30.8%). We found a difference in the predominant microbial etiology when we stratified patients with IE by DM. The predominant microorganism was *S. aureus* (36.5%) for patients with IE and DM, but this was *Streptococcus* spp. in patients without DM (29.4%), see Fig. 2.

Patients with IE and DM, who also used insulins, had an even higher proportion of *S. aureus* compared to patients with IE and DM who did not use insulins (43.2% vs. 32.3%), see Supplementary Fig. 1. The same pattern was observed when subdividing patients by type of DM, so that patients with Type 1 DM had a higher proportion of *S. aureus* compared to patients with Type 2 DM (48.1% vs. 32.3%), see Supplementary Fig. 2.

### In-hospital mortality

The in-hospital mortality was 22.4% in patients with DM compared to 18.0% in those without DM (p-value < 0.01). The associated HR of in-hospital mortality from the unadjusted model remained consistently higher for patients with IE and DM compared to those without DM (HR = 1.26 [95% CI: 1.11–1.43]), but the multivariable adjusted model was not significantly different (HR = 1.09 [95% CI: 0.95–1.24]), see Table 2.

**Table 2:** Estimates of mortality from regression models

		IE and DM	IE Without DM	
		HR (95% CI) ^a^	HR (95% CI) ^b^	*p*-value
In-Hospital Mortality^1^	*Unadjusted*	1.26 (1.11–1.43)	1.00 (ref.)	*p* < 0.001
	*Adjusted* ^*1*^	1.09 (0.95–1.24)	1.00 (ref.)	*p* = 0.217
One-Year Mortality from Admission^1^	*Unadjusted*	1.40 (1.27–1.54)	1.00 (ref.)	*p* < 0.001
	*Adjusted* ^*1*^	1.15 (1.04–1.27)	1.00 (ref.)	*p* = 0.006
One-Year Mortality from Discharge^2^	*Unadjusted*	1.64 (1.42–1.88)	1.00 (ref.)	*p* < 0.001
	*Adjusted* ^*1*^	1.26 (1.09–1.46)	1.00 (ref.)	*p* = 0.002

IE = Infective Endocarditis. DM = Diabetes Mellitus. HR = Hazard Ratio

^1^ Age (continuous scale), microbial etiology, sex, acute myocardial infarction, congestive heart failure, chronic obstructive lung disease, chronic kidney disease, chronic dialysis, malignancy, prior prosthetic heart valve, prior cardiac implantable electronic device

^2^ Age (categorical), microbial etiology, sex, acute myocardial infarction, congestive heart failure, chronic obstructive lung disease, chronic kidney disease, chronic dialysis, malignancy, prior prosthetic heart valve, prior cardiac implantable electronic device

### One-year mortality from admission

**Fig. 3:**
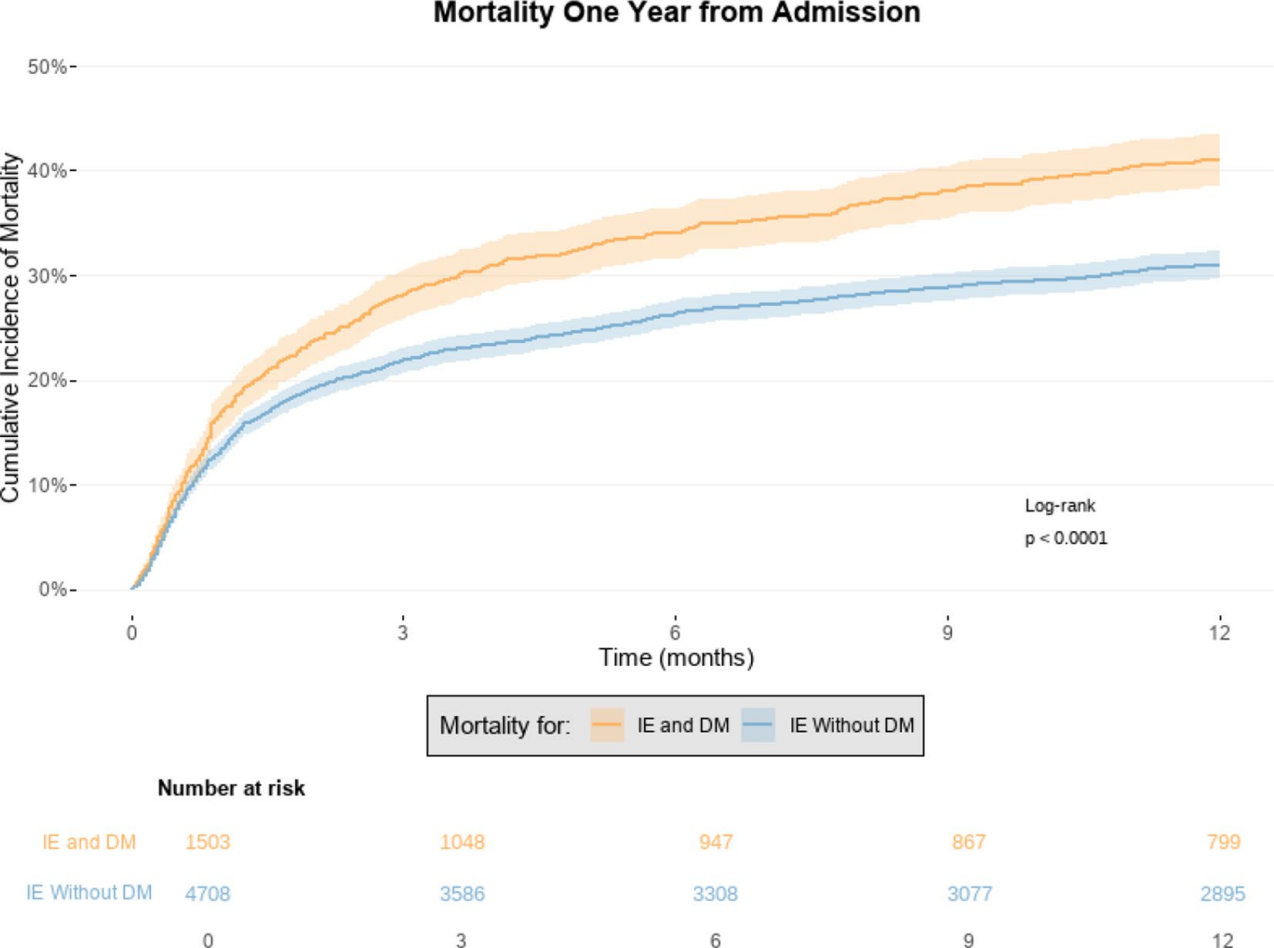
The figure shows the associated absolute risk of mortality with one year of follow-up from admission for patients with infective endocarditis (IE) and diabetes mellitus (DM) compared to those without DM. IE = Infective Endocarditis. DM = Diabetes Mellitus

The associated absolute mortality risk within one year from admission was higher for patients with IE and DM compared to those without DM (41.1% [95% CI: 38.5%-43.6%] vs. 31.0% [95% CI: 29.6%-32.3%], see Fig. 3.

The associated adjusted HR of one-year mortality from admission was significantly higher for patients with IE and DM compared to those without DM (HR = 1.15 [95% CI: 1.04–1.27]), see Table 2. The associated absolute risk of mortality within one year from admission was significantly higher for patients with IE and DM using insulins compared to those with DM who were not using insulins (44.8% [95% CI: 40.6%-48.9%] vs. 38.7% [95% CI: 35.5%-41.9%]), see Supplementary Fig. 3. The associated absolute risk of mortality within one year from admission was also significantly higher when subdividing patients with DM into Type 1 DM and Type 2 DM (45.3% [95% CI: 40.3%-50.2%] vs. 39.5% [95% CI: 36.6%-42.4%]), see Supplementary Fig. 4.

### One-year mortality from discharge

**Fig. 4:**
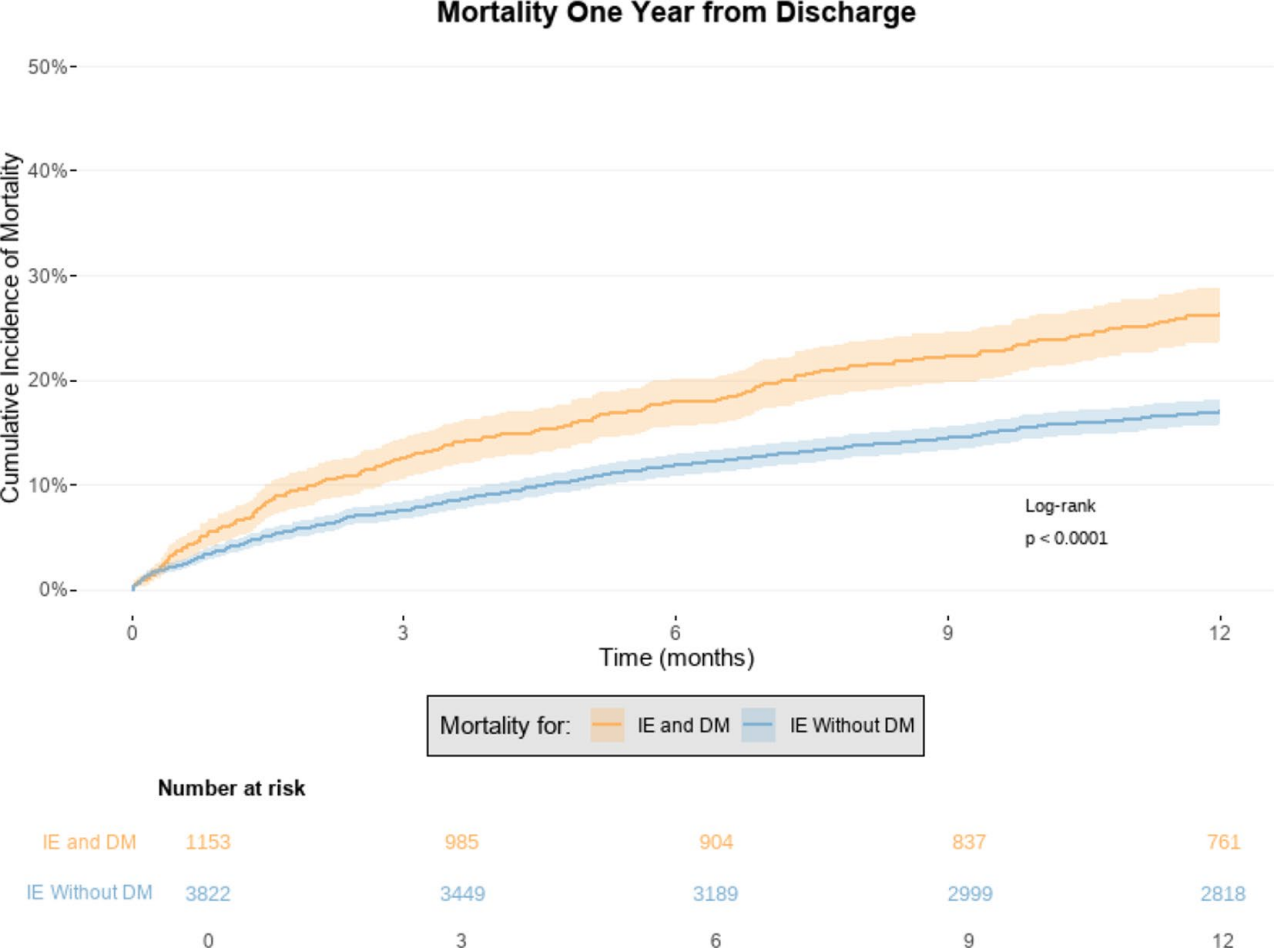
The figure shows the associated absolute risk of mortality with one year of follow-up in patients surviving their admission for first-time infective endocarditis (IE). Results are shown for patients with IE and diabetes mellitus (DM) compared to those without DM. IE = Infective Endocarditis. DM = Diabetes Mellitus

The number of patient alive on the day of discharge was 4,975 (80.0%). The associated absolute mortality risk within one year from discharge was higher for patients with IE and DM compared to those without DM (26.2% [95% CI: 23.7%-28.9%] vs. 16.9% [95% CI:15.7%-18.1%]), see Fig. 4.

The associated adjusted HR of mortality within one year from discharge was significantly higher for patients with IE and DM compared to patients without DM (HR = 1.26 [95% CI: 1.09–1.46]), see Table 2. This result remained robust when further adjusting for valve surgery during admission (HR = 1.25 [95% CI: 1.08–1.45]).

### One-year mortality for patients with valve surgery

The associated absolute mortality risk within one year from the date of surgery was higher for patients with IE and DM compared to those without DM (33.6% [95% CI: 27.2%-40.1%] vs. 15.3% [95% CI: 13.2%-17.6%]), see Supplementary Fig. 5. The patients with IE and DM who did not undergo valve surgery were associated with a higher absolute mortality risk within one year from admission compared to those without DM (42.5% [CI 95%: 39.7%-45.2%] vs. 35.6% [95% CI: 34.0%-37.2%]), see Supplementary Fig. 5.

### Analyses for the associated interactions between DM and age group

Patients with IE and DM who were younger than the age median (72.5 years) were associated with an increased in-hospital mortality and an increased mortality one year from admission, but the patients older than the age median were not (compared to the patients without DM), see Supplementary Table 4. The same pattern was observed for patients who survived their admission for IE (their age median was 71.6 years), see Supplementary Table 4.

## Discussion

In this nationwide, registry-based cohort study we examined the outcomes for patients with IE and DM compared to those without DM. We reported the patient characteristics, microbial etiologies, as well as the associated crude and adjusted mortality. Compared to those without DM, patients with IE and DM were more often male, were older, and had a higher burden of comorbidities. The most prominent differences in baseline characteristics were the proportion of chronic kidney disease and chronic dialysis that were substantially higher among patients with IE and DM. *S*. *aureus* was the most common infective bacteria amongst patients with IE and DM, and they were associated with an increased in-hospital mortality (unadjusted) and an increased one-year mortality from admission and from discharge (both adjusted and unadjusted) compared to those without DM.

### Proportion of diabetes mellitus and baseline characteristics

Only few studies have been performed on a nationwide scale for patients with IE. The current guidelines on the treatment of IE are largely based on observational studies from tertiary treatment centers with an inherit risk of referral bias [35]. We found 24% of patients with first-time IE to have DM. We previously found an increase in the proportion of DM for patients with IE from 11% to 22% (Danish nationwide data, 1997–2017) [3]. Other studies have reported the proportion of DM in patients with IE between 12 and 34% in Europe and North America [4–15]. Several of these studies have reported increasing proportions of DM among patients with IE [8–13]. However, our study found a relatively larger proportion of DM among patients with IE than previous nationwide studies and some of the studies from tertiary treatment centers. For instance, a nationwide registry-based study from Spain found 17% of patients with IE also presented with DM, and an American study covering New York and California found 15% of patients with IE presented with complicated DM [8, 9]. Also, results from the International Collaboration on Endocarditis-Prospective Cohort Study (ICE-PCS) showed that 16% of patients with IE also presented with DM. ICE-PCS consisted of 58 voluntarily participating tertiary treatment centers from 25 different countries. Compared to the current study, patients enrolled in ICE-PCS were generally younger while the age were comparable to both the Spanish and American study. A later Spanish study from 2016 to 2020 from the Grupo de Apoyo al Manejo de la Endocarditis Infecciosa en Espana (GAMES) cohort showed that the proportion of patients with IE who also presented with DM had increased to 27%. This indicates that the group of patients with IE and DM currently could be larger than previously expected. Studies from the last 20–30 years have reported a changing demographic of patients with IE towards higher proportion of males, age, and burden of comorbidities, which has also been the case in Denmark [18, 36]. In the current study, we found this to be more pronounced among patients with IE and DM compared to those without DM. Similar trends have been reported previously, so that patients with IE and DM have been reported as older, more often male, and presenting with an even higher burden of comorbidities [4, 5, 11, 12]. 

We found that patients with IE and DM were slightly older than those without DM. This may be explained by the fact that most patients in the current study presented with Type 2 DM, which typically develops later in life. Our results suggests that patient with IE and DM may be associated with a different risk-profile for IE compared to those without DM.

### Microbiology, kidney disease and dialysis

The diagnostic work-up for patients with IE includes blood-cultures, transthoracic echocardiography (TTE)/transoesophageal echocardiography (TOE), blood samples, medical history, and clinical assessment as the recommended treatment differs for various subgroups of patients with IE [1]. There are no specific recommendations for patients with IE and DM in the latest guidelines from the European Society of Cardiology, but there are recommendations for situations where IE is caused by *S. aureus* or when reduced kidney function is present [1]. During the last 20–30 years, *S. aureus* has become the predominant microbial etiology [16–18]. This has been linked to an increasing rate of prosthetic heart valve implantation or other invasive procedures. However, patients with DM have been associated with an increased risk of infections secondary to an altered or impaired immune function and diabetic foot ulcers that increases the risk of infections with *S. aureus* [20, 21]. We found *S. aureus* as the predominant microbial etiology in patients with IE and DM, but this was *Streptococcus* spp. in those without DM. This contrasts with some previous studies while others have found similar results, yet they were performed on specific subgroups of patients with IE and DM, were from tertiary treatment centers, or were based on voluntarily participating hospitals [5, 11, 12, 37]. DM is associated with increased occurrence of kidney disease [22]. Our study showed that this was also the case for patients with IE and DM as they had a higher proportion of both kidney disease and dialysis compared to those without DM. Both kidney disease and dialysis remains important comorbidities in patients with IE [1]. Previous studies have reported the proportion of kidney disease with significant variability in patients with IE and DM (7.9%-50.0%) and in those without DM (3.8%-34%) [5, 11, 12, 37]. The proportion of patients with IE and DM who are dependent on dialysis have rarely been reported and we add to the current knowledge with nationwide data. The differences in the proportions of kidney disease and dialysis may also constitute an important difference in the risk-profile and impact the associated outcomes for patients with IE and DM. Also, DM may be an important factor for the overall increase of *S. aureus* in patients with IE seen in the last 20–30 years, perhaps impacted by complex interactions that need further investigation.

### Mortality

The World Health Organization (WHO) estimated that 800 million people were living with DM in 2024 [38]. DM-associated mortality has decreased in western countries the last 20–30 years, yet their mortality remains higher compared to patients without DM [32, 39, 40]. Infections have been identified an important contributor to the excess mortality in patients with DM [19, 20]. 

Our study focused on patients with IE and DM, and we showed that these patients were associated with an increased in-hospital mortality (unadjusted) and an increased one-year from admission and from discharge (unadjusted and adjusted) compared to those without DM. Several other studies have found a higher in-hospital and one-year mortality in patients with IE and DM [4, 5, 11, 12, 15]. These studies were performed on various sub-groups of patients, or from tertiary treatment centers. This supports that DM may play an important role for patients with IE in various settings. We did not observe a significant difference in the adjusted in-hospital mortality in the present study. Many factors could contribute to this, but it suggests that other comorbidities than DM also impact the in-hospital mortality in the acute setting of IE. We found an interaction between age group and DM for all primary mortality outcomes for patients with IE. We believe this pattern was due to an increasing importance of other comorbidities than DM with increasing age (competing risk). To the best of our knowledge no previous studies have reported the one-year mortality from discharge and we delivered novel data on that outcome-measure.

We speculate if the mortality of patients with IE is impacted more when DM is present, than previously expected. However, patients with IE and DM are also associated with a significantly higher burden of comorbidities and these may also play an important role, particularly in relation to in-hospital mortality and amongst the oldest patients. We advise that patients with DM should be given special attention when admitted for IE and that future research should evaluate potential high-risk subgroups within this population.

### Strengths and limitations

We consider the following valuable strengths. We used a validated approach of defining the IE diagnosis with a PPV of 90%. The Danish registries are of high quality with nationwide coverage that limits the risk of referral bias, and they ensure comprehensive follow-up on an individual level. We consider the following as important limitations. To the authors knowledge no studies have assessed the PPV of DM from Danish registries and our definition may have led to some misclassification. No information on echocardiography or hemoglobin A1C levels were available. Danish registries do not have information available to distinguish between definite or possible IE. Residual confounding from unmeasured effects or unknown variables may be present. Our study was based on large administrative registries, and detailed information on the sources of the infective organisms was not available. Our study was an observational study, and we could only report associations rather than causal effects.

## Conclusion

This nationwide study had three main findings: (1) Patients with IE and DM present with a higher burden of comorbidities, (2) the most predominant infective microorganism was *S. aureus* for patients with DM, but this was *Streptococcus* spp. for those without DM, (3) patients with DM were associated with a higher mortality compared to those without DM.

Our findings emphasize the need for further research in this clinically important patient group with more severe outcomes and they call for improved management of IE patients with DM.

## Electronic supplementary material

Below is the link to the electronic supplementary material.

Supplementary Material 1

## Data Availability

No datasets were generated or analysed during the current study.
