# Supplementary material for: Infective endocarditis and relationship with diabetes mellitus - Patient characteristics, microbial etiology and mortality
Source: Infection. 2025 Sep 10;53(6):2757–67. doi: 10.1007/s15010-025-02624-7 (PMC12675667; doi:10.1007/s15010-025-02624-7)
Supplement: Supplementary file 1 — Supplementary Material 1 [file 15010_2025_2624_MOESM1_ESM.docx]

**Supplementary Figure Legends**

Supplementary Fig. 1: The figure shows the microbial etiology in patients with infective endocarditis (IE) and diabetes mellitus (DM) who also use insulins, and patients with IE and DM who do not use insulins (compared to those without DM). IE = Infective Endocarditis. DM = Diabetes Mellitus, CoNS = Coagulase-Negative Staphylococci.

Supplementary Fig. 2: The figure shows the microbial etiology in patients with infective endocarditis (IE) and diabetes mellitus (DM) when subdividing by type of DM. IE = Infective Endocarditis. DM = Diabetes Mellitus, CoNS = Coagulase-Negative Staphylococci.

Supplementary Fig. 3: The figure shows the associated absolute risk of mortality with one year of follow-up for patients with infective endocarditis (IE) and diabetes mellitus (DM) using insulins, for patients with IE and DM not using insulins (compared to those without DM). IE = Infective Endocarditis. DM = Diabetes Mellitus.

Supplementary Fig. 4: The figure shows the associated absolute risk of mortality with one year of follow-up for patients with infective endocarditis (IE) and diabetes mellitus (DM) when subdividing by Type 1 DM and Type 2 DM (compared to those without DM). IE = Infective Endocarditis. DM = Diabetes Mellitus.

Supplementary Fig. 5: The figure shows the associated absolute risk of mortality for patients with infective endocarditis (IE) and diabetes mellitus (DM) compared to those without DM when they were also grouped by valve surgery during admission. The follow up was up to one year. Patients undergoing valve surgery were followed from the day of surgery, and all other patients were followed from the day of admission. IE = Infective Endocarditis. DM = Diabetes Mellitus.

**Supplementary Table 1: Codes Used**

| *ICD, NOMESCO, and ATC codes used* | | | |
| --- | --- | --- | --- |
| **Category** | | **Type** | **Codes** |
| **Study population:** | |  |  |
| Infective Endocarditis | |  |  |
| - In-hospital diagnosis code | | ICD-10 | I33.x, I38.x, I39.8 |
|  |  | ICD-8 | 421 |
| Diabetes Mellitus (DM) (any of below) | |  |  |
| - In-hospital or out-patient diagnosis code prior to admission for infective endocarditis - Use of antidiabetic medication within 6-months of admission for infective endocarditis. | | ICD-10 ICD-8  ATC | E10.x to E14.x  249.x, 250.x  A10A.x (insulins), A10B.x (non-insulins) |
|  | Type 1 DM (both of below) |  |  |
|  | - An ICD-10 code of Type 1 DM prior to admission for infective endocarditis - Use of insulins within 6 months of admission for infective endocarditis. | ICD-10  ATC | E10.x  A10A.x (insulins) |
|  | Type 2 DM (both of below) |  |  |
|  | - Classified as a patient with DM - Not classified as patient with Type 1 DM |  |  |
| Diabetes Mellitus and Use of Insulin (both of below) | |  |  |
| - In-hospital or out-patient diagnosis code prior to admission for infective endocarditis - Use of insulins within 6 months of admission for infective endocarditis. | | ICD-10  ICD-8  ATC | E10.x to E14.x  249.x, 250.x  A10A.x (insulins) |
| **Medical history prior to admission:** | |  |  |
| Acute Myocardial Infarction | | ICD-10 | I21.x, I22.x |
|  | | ICD-8 | 410 |
| Alcohol Abuse | | ICD-10 | E244, E529A, F10.x, G312, G621, G721, I426, K292, K70.x, K860, L278A, O354, Z714, Z721 |
|  | | ICD-8 | 291, 303, 571.x, 57710 |
|  | |  |  |
| Aortic Valve Disease | | ICD-10 | I35.x |
|  | | ICD-8 | 395, 396 |
| Atrial Fibrillation/flutter | | ICD-10 | I48.x |
|  | | ICD-8 | 42793, 42794 |
| Chronic Obstructive Lung Disease | | ICD-10 | J43.x, J44.x |
|  |  | ICD-8 | 492 |
| Chronic Kidney Disease (any of below) | |  |  |
| - Chronic Kidney Disease (see below for dialysis) | | ICD-10 | E10.2, E11.2, E13.2, E14.2, I12.0, I13.x,  N02.x, N03.x, N07.x, N08.x, N11.x, N12.x, N14.x, N15.8, N15.9, N16.0, N16.2 to N16.4, N16.8, N18.x, N19.x, N26.x, Q61.1, Q61.2, Q61.3, Q61.5, Q61.9 |
|  | | ICD-8 | 25002, 403, 404, 40039, 582, 584, 59009, 59320, 75311, 75319 |
| - Dialysis When Chronic Kidney Disease | | NOMESCO | BJFD2 |
| Congestive Heart Failure | | ICD-10 | I11.0, I13.0, I13.2, I50.x |
|  | | ICD-8 | 42709, 42719 |
| Hypertension | | ICD-10 | I109 |
|  | | ICD-10 | 400.x, 401.x, 402.x, 403.x, 404.x |
| Ischemic/Hemorrhagic Stroke | | ICD-10 | I60.x to I64.x |
|  | | ICD-8 | 430 to 436 |
| Liver disease | | ICD-10 | B16.x to B19.x, D68.4C I98.2, K70.x to K77.0, Q61.8A, Z94.4 |
|  | | ICD-8 | 070, 155, 571 to 573 |
| Malignancy | | ICD-10 | C00.x to C97.x (not C44.x) |
|  |  | ICD-8 | 140 to 209 (not 173.x) |
| Mitral Valve Disease | | ICD-10 | I34.x |
|  | | ICD-8 | 394, 396 |
| **Surgery or procedures:** | |  |  |
| Cardiac Implantable Electronic Devices | | NOMESCO | BFCA0, BFCB0 |
| Dialysis When Chronic Kidney Disease | | NOMESCO | BJFD2 |
| Prosthetic Heart Valve | | NOMESCO | KFMD.x, KFKD.x, KFGE.x, KFJF.x, KFCA60, KFCA70 |
| Valve Surgery | | NOMESCO | KFM.x, KFG.x, KFK.x, KFJ.x, KFCA60, KFCA70 |
| **Pharmacotherapy prior to admission:** | |  |  |
| Aspirin | | ATC | B01AC06 |
| Adenosine Diphosphate Receptor Inhibitors | | ATC | B01AC04, B01AC22, B01AC24 |
| Antihypertensive Medication | | ATC | C02A.x, C02B.x, C02C.x, C02DA.x, C02DB.x, C02DD.x, C02DG.x, C02L.x, C03A.x, C03B.x, C03D.x, C03E.x, C03X.x, C04.x, C05.x, C07.x, C08.x, C09.x |
| Betablockade | | ATC | C07.x, C09BX02, C09BX04, C09BX05, C09BX09 |
| Medication Used in Alcohol Dependence | | ATC | N07BB.x |
| Loop Diuretics | | ATC | C03C.x, C03EB01, C03EB02 |
| NSAID | | ATC | M01A.x |
| Glucocorticoids | | ATC | H02A.x, H02B.x |
| Vitamin K antagonists | | ATC | B01AA.x |
| Statins | | ATC | C10AA.x |
| Antidiabetic Medication | | ATC | A10A.x (insulins)  A10B.x (non-insulins)   - Of them: A10BJ (Glucagon-Like Peptide 1 Agonists) - Of them: A10BK (Sodium-Glucose Co-Transporter 2 Inhibitors) |
| “.x” indicates every sub-classification of diagnosis code or ATC code.  ICD = International Classification of Diseases.  NOMESCO = The Nordic Medico-Statistical Committee Classification of Surgical Procedures.  ATC = Anatomical Therapeutic Chemical Classification System.  NSAID = Non-steroidal Anti-inflammatory Drugs | | | |

**Supplementary Table 2: Baseline Characteristics in Patients with IE Grouped by Diabetes Mellitus and use of Insulins**

|  | | **First-time IE 2010–2020**  N = 6,211 (100%) | | |  |
| --- | --- | --- | --- | --- | --- |
|  | | **IE and DM With Insulins**  N = 576 (9.3%) | **IE and DM Without Insulins**  N = 927 (14.9%) | **IE Without DM**  (N = 4,708) | p-value |
| **Demographics at Baseline, N (%)** | | | | | |
| Male | | 377 (65.5%) | 646 (69.7%) | 3,155 (67.0%) | 0.18 |
| Age | | 71.7 (63.7, 78.2) | 73.2 (66.7, 79.8) | 72.4 (61.5, 80.5) | < 0.01 |
| Age Group | |  |  |  | < 0.01 |
|  | < 70 Years | 246 (42.7%) | 316 (34.1%) | 2,056 (43.7%) |  |
|  | ≥ 70 Years | 330 (57.3%) | 611 (65.9%) | 2,652 (56.3%) |  |
| **Hospitalization and Procedures, N (%)** | | | | | |
| Days Admitted | | 36.0 (25.0, 49.0) | 38.0 (25.0, 50.0) | 38.0 (28.0, 49.0) | 0.60 |
| Valve Surgery During Admission | | 60 (10.4%) | 153 (16.5%) | 1,062 (22.6%) | < 0.01 |
| **Comorbidities at Baseline, N (%)** | | | | | |
| Type 1 DM | | 393 (68.2%) | 0 (0.0%) | 0 (0.0%) | < 0.01 |
| Acute Myocardial Infarction | | 131 (22.7%) | 171 (18.4%) | 473 (10.0%) | < 0.01 |
| Congestive Heart Failure | | 216 (37.5%) | 302 (32.6%) | 863 (18.3%) | < 0.01 |
| Atrial Fibrillation/flutter | | 211 (36.6%) | 390 (42.1%) | 1,219 (25.9%) | < 0.01 |
| Disease of The Aortic Valve | | 164 (28.5%) | 317 (34.2%) | 1,440 (30.6%) | 0.04 |
| Prosthetic Heart Valve Prior to Admission | | 121 (21.0%) | 210 (22.7%) | 962 (20.4%) | 0.31 |
| CIED Prior to Admission | | 156 (27.1%) | 210 (22.7%) | 733 (15.6%) | < 0.01 |
| Ischemic/hemorrhagic Stroke | | 111 (19.3%) | 164 (17.7%) | 587 (12.5%) | < 0.01 |
| Chronic Obstructive Lung Disease | | 101 (17.5%) | 183 (19.7%) | 521 (11.1%) | < 0.01 |
| Liver Disease | | 61 (10.6%) | 92 (9.9%) | 320 (6.8%) | < 0.01 |
| Malignancy | | 110 (19.1%) | 176 (19.0%) | 903 (19.2%) | 0.99 |
| Chronic Kidney Disease | | 293 (50.9%) | 246 (26.5%) | 521 (11.1%) | < 0.01 |
| Dependent on Dialysis | | 128 (22.2%) | 113 (12.2%) | 262 (5.6%) | < 0.01 |
| Hypertension | | 450 (78.1%) | 702 (75.7%) | 2,198 (46.7%) | < 0.01 |
| **Pharmacotherapy Within 6 Months Prior to Admission, N (%)** | | | | | |
| Statin | | 378 (65.6%) | 559 (60.3%) | 1,530 (32.5%) | < 0.01 |
| Aspirin | | 245 (42.5%) | 329 (35.5%) | 1,114 (23.7%) | < 0.01 |
| NSAID | | 78 (13.5%) | 149 (16.1%) | 824 (17.5%) | 0.04 |
| Corticosteroids | | 91 (15.8%) | 112 (12.1%) | 545 (11.6%) | 0.01 |
| Use of Insulins | | 576 (100.0%) | 0 (0.0%) | 0 (0.0%) | < 0.01 |
| Use of SGLT2-inhibitors or GLP-1 agonists | | 72 (12.5%) | 65 (7.0%) | < 4 (< 0.1%) | < 0.01 |
| Use of other antidiabetic medication | | 213 (37%) | 597 (64.4%) | 0 (0.0%) | < 0.01 |
| IE = Infective Endocarditis, DM = Diabetes Mellitus, CIED = Cardiac Implantable Electronic Device, NSAID = Non-Steroidal Anti-Inflammatory Drug  Categorical variables presented as count and percent.  Continuous variables presented as the median with corresponding 25th to P75th percentile. | | | | | |

|  | | **First-time IE 2010–2020**  N = 6,211 (100%) | | |  |
| --- | --- | --- | --- | --- | --- |
|  | | **IE and Type 1 DM**  N = 393 (6.3%) | **IE and Type 2 DM**  (N = 1,110) | **IE Without DM**  (N = 4,708) | P value |
| **Demographics at Baseline, N (%)** | | | | | |
| Male | | 255 (64.9%) | 768 (69.2%) | 3,155 (67.0%) | 0.22 |
| Age | | 70.9 (61.5, 77.5) | 73.5 (66.7, 79.8) | 72.4 (61.5, 80.5) |  |
| Age Group | |  |  |  | < 0.01 |
|  | < 70 Years | 185 (47.1%) | 377 (34.0%) | 2,056 (43.7%) |  |
|  | ≥ 70 Years | 208 (52.9%) | 733 (66.0%) | 2,652 (56.3%) |  |
| **Hospitalization and Procedures, N (%)** | | | | | |
| Days Admitted | | 38.0 (27.0, 50.0) | 37.0 (25.0, 49.0) | 38.0 (28.0, 49.0) |  |
| Valve Surgery During Admission | | 36 (9.2%) | 177 (15.9%) | 1,062 (22.6%) | < 0.01 |
| **Comorbidities at Baseline, N (%)** | | | | | |
| Type 1 DM | | 393 (100.0%) | 0 (0.0%) | 0 (0.0%) | < 0.01 |
| Acute Myocardial Infarction | | 94 (23.9%) | 208 (18.7%) | 473 (10.0%) | < 0.01 |
| Congestive Heart Failure | | 153 (38.9%) | 365 (32.9%) | 863 (18.3%) | < 0.01 |
| Atrial Fibrillation/flutter | | 135 (34.4%) | 466 (42.0%) | 1,219 (25.9%) | < 0.01 |
| Disease of The Aortic Valve | | 101 (25.7%) | 380 (34.2%) | 1,440 (30.6%) | < 0.01 |
| Prosthetic Heart Valve Prior to Admission | | 74 (18.8%) | 257 (23.2%) | 962 (20.4%) | 0.08 |
| CIED Prior to Admission | | 99 (25.2%) | 267 (24.1%) | 733 (15.6%) | < 0.01 |
| Ischemic/hemorrhagic Stroke | | 83 (21.1%) | 192 (17.3%) | 587 (12.5%) | < 0.01 |
| Chronic Obstructive Lung Disease | | 77 (19.6%) | 207 (18.6%) | 521 (11.1%) | < 0.01 |
| Liver Disease | | 40 (10.2%) | 113 (10.2%) | 320 (6.8%) | < 0.01 |
| Malignancy | | 76 (19.3%) | 210 (18.9%) | 903 (19.2%) | 0.98 |
| Chronic Renal Disease | | 223 (56.7%) | 316 (28.5%) | 521 (11.1%) | < 0.01 |
| Dependent on Dialysis | | 110 (28.0%) | 131 (11.8%) | 262 (5.6%) | < 0.01 |
| Hypertension | | 313 (79.6%) | 839 (75.6%) | 2198 (46.7%) | < 0.01 |
| Statin | | 257 (65.4%) | 680 (61.3%) | 1530 (32.5%) | < 0.01 |
| Aspirin | | 180 (45.8%) | 394 (35.5%) | 1114 (23.7%) | < 0.01 |
| NSAID | | 53 (13.5%) | 174 (15.7%) | 824 (17.5%) | 0.06 |
| Corticosteroids | | 53 (13.5%) | 150 (13.5%) | 545 (11.6%) | 0.13 |
| Use of Insulins | | 393 (100.0%) | 183 (16.5%) | 0 (0.0%) | < 0.01 |
| Use of SGLT2-inhibitors or GLP-1 agonists | | 35 (8.9%) | 102 (9.2%) | < 4 (< 0.1%) | < 0.01 |
| Use of other antidiabetic medication | | 106 (27.0%) | 704 (63.4%) | 0 (0.0%) | < 0.01 |
| IE = Infective Endocarditis, DM = Diabetes Mellitus, CIED = Cardiac Implantable Electronic Device, NSAID = Non-Steroidal Anti-Inflammatory Drug  Categorical variables presented as count and percent.  Continuous variables presented as the median with corresponding 25th to P75th percentile. | | | | | |

**Supplementary Table 4: The Associated Estimates of Mortality for Patients with IE and DM when Split by Age Group**

|  | | In-Hospital Mortality^2^ | | | One-Year Mortality from Admission^2^ | | | One-Year Mortality from Discharge^2^ | | |
| --- | --- | --- | --- | --- | --- | --- | --- | --- | --- | --- |
|  |  | *HR*  *(95% CI) ^a^* | *Crude estimates* | | *HR*  *(95% CI) ^a^* | *1-KM estimates of mortality*  *(in %)* | | *HR*  *(95% CI) ^a^* | *1-KM estimates of mortality*  *(in %)* | |
| **Under the Age Median^1^** | IE and DM | 1.46 (1.18–1.81) | | 20.6% | 1.55 (1.31–1.83) | | 36.1 (32.6–39.6) | 1.66 (1.28–2.15) | | 21.6 (18.1–25.2) |
|  | IE Without DM | REF | | 12.2% | REF | | 20.3 (18.7–22.0) | REF | | 10.1 (8.8-  11.5) |
| **Over the Age Median^1^** | IE and DM | 0.92 (0.77–1.10) | | 23.7% | 0.97 (0.86–1.11) | | 45.9 (42.3–49.5) | 1.10 (0.92–1.32) | | 30.3 (26.7–34.0) |
|  | IE Without DM | REF | | 23.5% | REF | | 41.8 (39.8–43.8) | REF | | 24.1 (22.2–26.2) |
| IE = Infective Endocarditis. DM = Diabetes Mellitus. HR = Hazard Ratio. 1-KM = Inverse Kaplan-Meier Estimates  ^1^ The overall age median was 72.5 years when following patients from admission. The age median was 71.6 years for patients surviving their admission for IE.  ^2^ Adjusted for: Microbial etiology, sex, acute myocardial infarction, congestive heart failure, chronic obstructive lung disease, chronic kidney disease, chronic dialysis, malignancy, prior prosthetic heart valve, prior cardiac implantable electronic device. | | | | | | | | | | |

**Supplementary Fig. 1: Microbial Etiology in Patients with Infective Endocarditis Grouped by Diabetes Mellitus and use of Insulins**

**
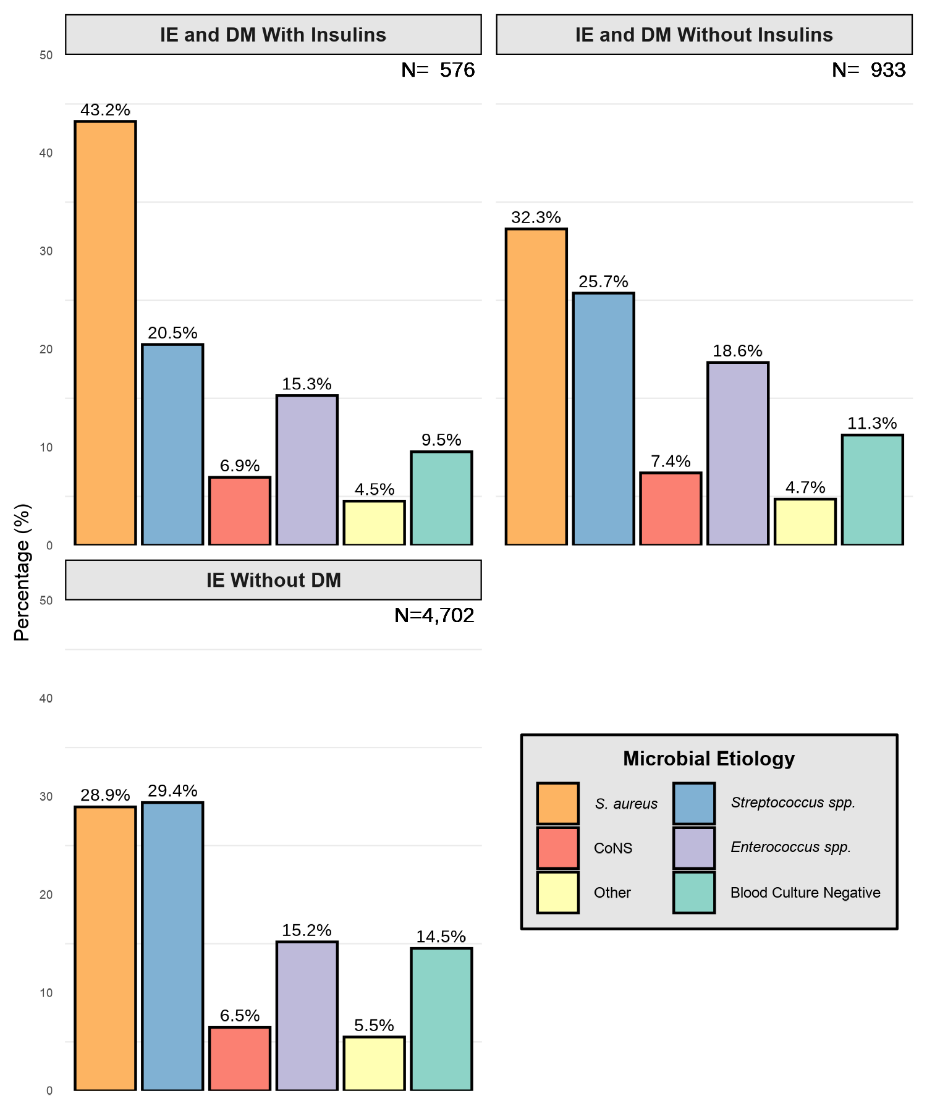
**

Supplementary Fig. 1: The figure shows the microbial etiology in patients with infective endocarditis (IE) and diabetes mellitus (DM) who also use insulins, and patients with IE and DM who do not use insulins (compared to those without DM). IE = Infective Endocarditis. DM = Diabetes Mellitus, CoNS = Coagulase-Negative Staphylococci.

**Supplementary Fig. 2: Microbial Etiology in Patients with Infective Endocarditis Subdivided by Type of Diabetes**


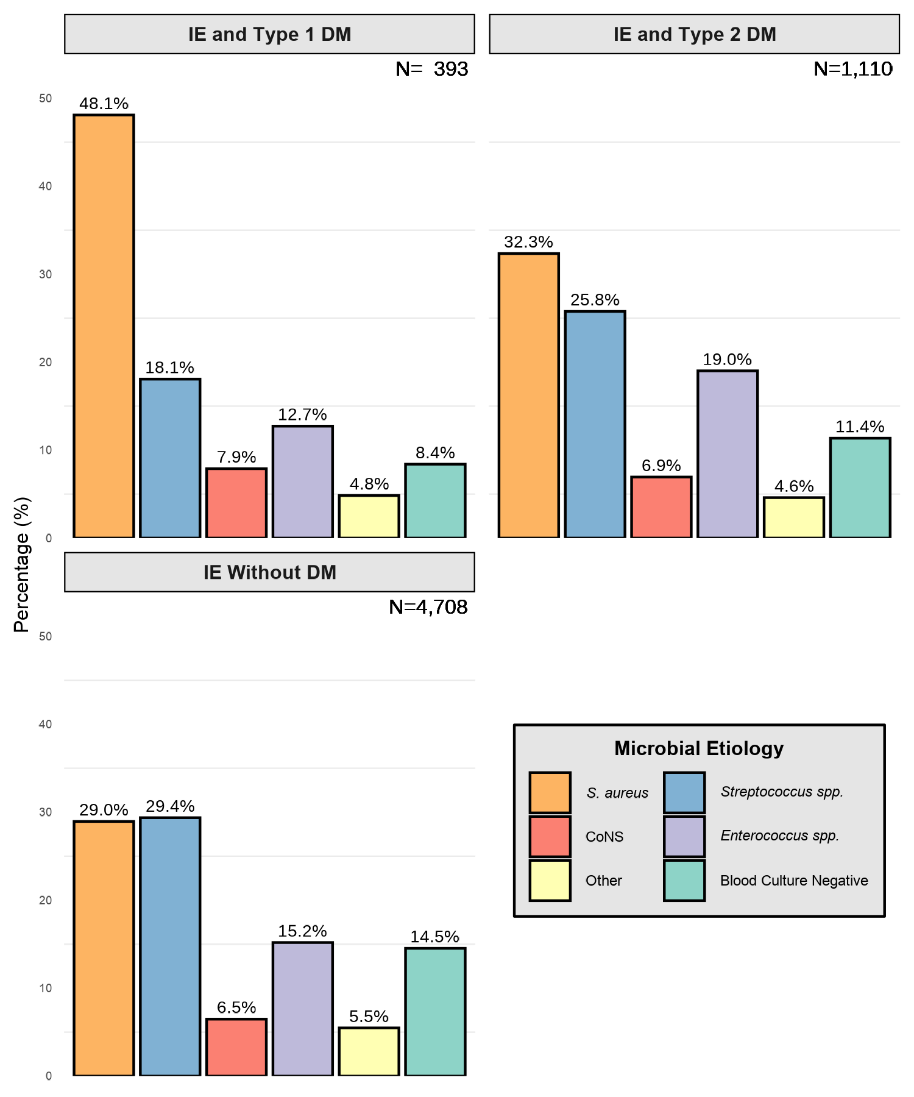


Supplementary Fig. 2: The figure shows the microbial etiology in patients with infective endocarditis (IE) and diabetes mellitus (DM) when subdividing by type of DM. IE = Infective Endocarditis. DM = Diabetes Mellitus, CoNS = Coagulase-Negative Staphylococci.

**Supplementary Fig. 3: Absolute Risk of One-year Mortality When Accounting for Use of Insulins**


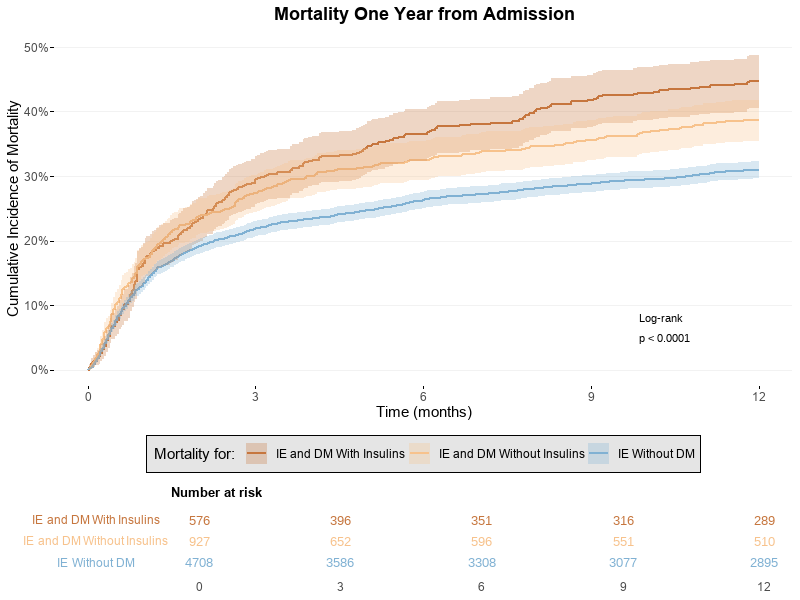


Supplementary Fig. 3: The figure shows the associated absolute risk of mortality with one year of follow-up for patients with infective endocarditis (IE) and diabetes mellitus (DM) using insulins, for patients with IE and DM not using insulins (compared to those without DM). IE = Infective Endocarditis. DM = Diabetes Mellitus.

**Supplementary Fig. 4: Absolute Risk of One-year Mortality When Subdividing by Type of Diabetes**


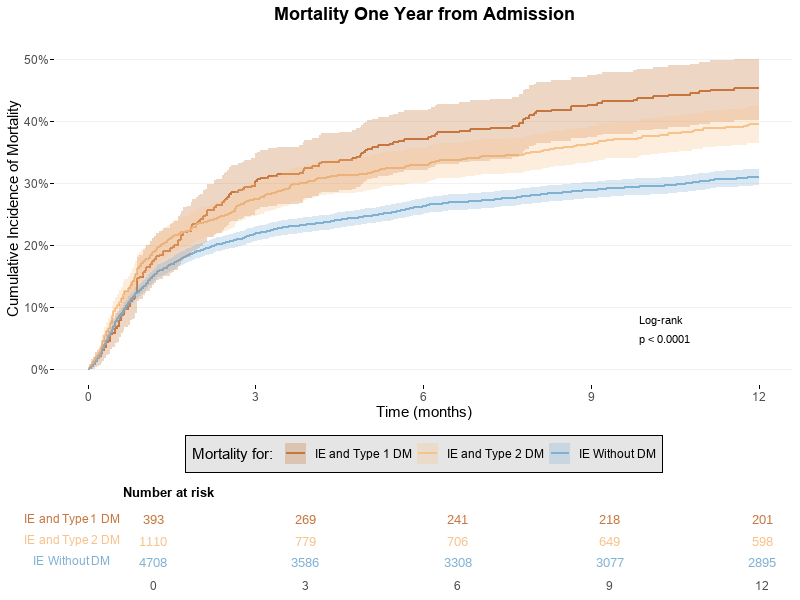


Supplementary Fig. 4: The figure shows the associated absolute risk of mortality with one year of follow-up for patients with infective endocarditis (IE) and diabetes mellitus (DM) when subdividing by Type 1 DM and Type 2 DM (compared to those without DM). IE = Infective Endocarditis. DM = Diabetes Mellitus.

**Supplementary Fig. 5: Absolute Risk of One-year Mortality When Accounting for Valve Surgery**


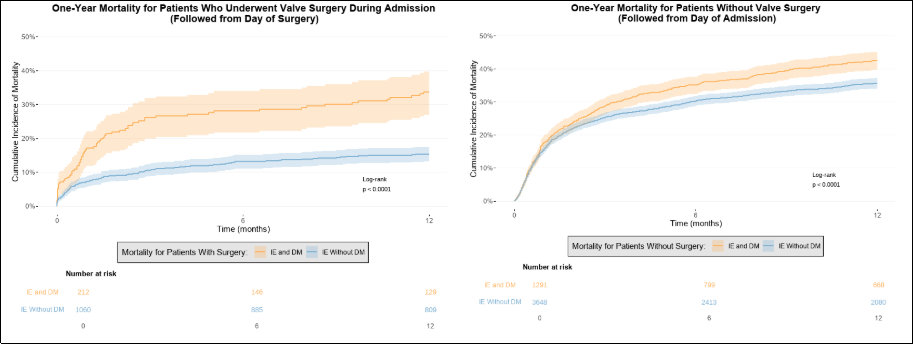


Supplementary Fig. 5: The figure shows the associated absolute risk of mortality for patients with infective endocarditis (IE) and diabetes mellitus (DM) compared to those without DM when they were also grouped by valve surgery during admission. The follow up was up to one year. Patients undergoing valve surgery were followed from the day of surgery, and all other patients were followed from the day of admission. IE = Infective Endocarditis. DM = Diabetes Mellitus.
